# Long-Term Glucose Starvation Induces Inflammatory Responses and Phenotype Switch in Primary Cortical Rat Astrocytes

**DOI:** 10.1007/s12031-021-01800-2

**Published:** 2021-02-12

**Authors:** Vanessa Kogel, Stefanie Trinh, Natalie Gasterich, Cordian Beyer, Jochen Seitz

**Affiliations:** 1 Institute of Neuroanatomy, RWTH Aachen University, 52074 Aachen, Germany; 2 Department of Child and Adolescent Psychiatry, Psychosomatics and Psychotherapy, University Hospital, RWTH Aachen University, 52074 Aachen, Germany

**Keywords:** Anorexia nervosa, Astrocytes, Glucose deprivation, Astrogliosis, UPR, Neuroinflammation

## Abstract

Astrocytes are the most abundant cell type in the brain and crucial to ensure the metabolic supply of neurons and their synapse formation. Overnutrition as present in patients suffering from obesity causes astrogliosis in the hypothalamus. Other diseases accompanied by malnutrition appear to have an impact on the brain and astrocyte function. In the eating disorder anorexia nervosa (AN), patients suffer from undernutrition and develop volume reductions of the cerebral cortex, associated with reduced astrocyte proliferation and cell count. Although an effect on astrocytes and their function has already been shown for overnutrition, their role in long-term undernutrition remains unclear. The present study used primary rat cerebral cortex astrocytes to investigate their response to chronic glucose starvation. Cells were grown with a medium containing a reduced glucose concentration (2 mM) for 15 days. Long-term glucose starvation increased the expression of a subset of pro-inflammatory genes and shifted the primary astrocyte population to the pro-inflammatory A1-like phenotype. Moreover, genes encoding for proteins involved in the unfolded protein response were elevated. Our findings demonstrate that astrocytes under chronic glucose starvation respond with an inflammatory reaction*.* With respect to the multiple functions of astrocytes, an association between elevated inflammatory responses due to chronic starvation and alterations found in the brain of patients suffering from undernutrition seems possible.

## Introduction

Astrocytes are the most abundant cell type in the brain and fulfill many tasks to ensure physiological processes in the healthy brain. These include the physical and metabolic support of neurons as well as the reaction to injury of the central nervous system and synapse formation and modulation (Sofroniew and Vinters 2010). After brain injury or during neurological diseases, astrocytes undergo a transformation into a so-called reactive state resulting in astrogliosis (Liddelow and Barres 2017). Primarily, astrogliosis is a protective response to handle acute stress, to limit tissue damage and to restore brain homeostasis (Myer et al. 2006). This is achieved by glutamate uptake, free radical scavenging, neurotrophin release, and by reducing the damage induced by inflammatory reactions to a restricted area by scar formation (Chen and Swanson 2003). Extensive scar formation can, however, inhibit axon regeneration and other adaptive mechanisms to recover proper physiological functions (Zhang and Barres 2010). Astrogliosis is accompanied by altered expression of a set of genes, for example glial fibrillary acidic protein (GFAP) or several cytokines as well as by morphological and functional changes of astrocytes (Sofroniew 2014). Reactive astrocytes can be differentiated into at least two phenotypes (Liddelow et al. 2017; Zamanian et al. 2012). An A1-like astrocyte appears pro-inflammatory and can be toxic to neurons and oligodendrocytes; an A2-like astrocyte is neuroprotective, upregulates neurotrophic genes, and promotes neuronal survival (Liddelow and Barres 2017).

Another important function of astrocytes is to guarantee the metabolic supply of neurons and the regulation of glucose uptake into cerebral microvessels (Kacem et al. 1998) while sensing changes of glucose levels of the brain (Allard et al. 2014). Neurons need high levels and a constant supply with glucose to maintain their proper metabolic function (Mergenthaler et al. 2013). Astrocytes can store small amounts of glycogen which they, if necessary, break down to glucose and metabolize to lactate (Falkowska et al. 2015). To fulfill this functional characteristic, astrocytes are highly metabolically flexible and can rapidly upregulate glycolysis. In the event of an undersupply, astrocytes thus ensure the survival and function of neurons by providing lactate (Kasischke et al. 2004; Pellerin and Magistretti 1994).

It was recently shown by using rat models for anorexia nervosa (AN) that undernutrition has a clear impact on astrocytes and, in consequence, potentially on brain homeostasis (Frintrop et al. 2018, 2019; Reyes-Haro et al. 2016). Another study has presented evidence that overnutrition in patients suffering from obesity leads to micro- and astrogliosis in the hypothalamus (Thaler et al. 2012). A link between astrocytes, inflammatory reactions, and the progression of the respective eating disorder appears among other factors likely, since the hypothalamus is important for coordinating satiety and hunger (Bruch 1993; García-Cáceres et al. 2019).

A dysregulation of the unfolded protein response (UPR), for example in astrocytes, is also known to play a role in the development of metabolic diseases like obesity and diabetes (Feng et al. 2009; Yang et al. 2017). UPR is needed to prevent an accumulation of misfolded proteins in the endoplasmic reticulum (ER); otherwise, a condition known as ER stress is initiated (Hetz 2012). It is involved in the regulation of the glucose metabolism by switching the metabolic activity of the mitochondria from glucose to lipid metabolism (Piperi et al. 2016). The lack of UPR-associated activation transcription factor 6 (ATF6) increases neuronal cell death after brain ischemia probably due to a reduced astrocyte activation (Yoshikawa et al. 2015). In drosophila melanogaster, the UPR is believed to shift the metabolism from oxidative phosphorylation to glycolysis via the activating transcription factor 4 (ATF4) (Lee et al. 2015). These data suggest a close relationship between the UPR, metabolic activity, and diseases associated with malnutrition.

AN is a psychiatric illness characterized by a severe undernutrition leading to an insufficient energy intake (American Psychiatric Association 2013; Herpertz-Dahlmann 2015). It is often accompanied by mild hypoglycemia, which is usually asymptomatic (Mattingly and Bhanji 1995). As a consequence of starvation, brain volume loss was found in patients with AN (Seitz et al. 2014, 2016) linked to neuropsychological and learning deficits (Buehren et al. 2011; Castro-Fornieles et al. 2010). Moreover, brain volume loss of the cerebral cortex and corpus callosum (CC) was shown by our group in an experimental animal model for AN called activity-based anorexia (ABA). The observed brain volume reduction was accompanied by a decrease in GFAP-positive astrocytes as well as by reduced GFAP mRNA levels (Frintrop et al. 2018, 2019). Previous studies had already shown reduced learning in ABA animals after chronic starvation (Paulukat et al. 2016). Despite these findings, there is only sparse information available on alterations of the astrocytes themselves and which functional consequences these alterations and reduced numbers of astroglia may have on brain and synaptic physiology in the ABA rat model and patients with AN.

In this study, we aimed at establishing an in vitro model to investigate the effects of long-term glucose semi-starvation on primary rat cerebral cortex–derived astrocytes. In contrast to previous studies dealing with acute starvation ranging from 30 min to 48 h (Hara et al. 1989; Lee et al. 2016; Pauwels et al. 1985), a chronic model of semi-starvation lasting for 15 days will be used. Moreover, our research strategy goes far deeper into the cellular aspects and responses of starvation than the previous studies. This relates to our recent in vivo studies using an ABA rat model of starvation, where we could demonstrate that cerebral cortex astrocytes are mainly reduced under chronic starvation conditions and thus potentially contribute to brain volume loss as shown by MRI analysis (Frintrop et al. 2018, 2019)*.* Since astrogliosis is a common feature of metabolic diseases associated with malnutrition, we specifically analyzed the expression of genes associated with the A1 and A2 phenotype, pro-inflammatory pathways, and UPR.

## Methods

### Primary Cell Cultures

All animals used in this study were acquired and cared for in accordance with the Federation of European Laboratory Associations (FELASA) recommendations. Primary astrocyte cultures were obtained from the cerebral cortex and adjacent white matter of 1–3-day-old Wistar rat pups (Janvier Labs, France). Briefly, the pups were decapitated, the brains dissected, and the meninges removed. The tissues were incubated in ice-cold HEPES buffer and homogenized mechanically in 10 mL ice-cold Dulbecco’s modified Eagle’s medium (DMEM; 41966029, GibcoTM, Thermo Fisher Scientific, Waltham, MA, USA) supplemented with 10% fetal calf serum (10270-106, GibcoTM, Thermo Fisher Scientific, Waltham, MA, USA), 50 U/mL penicillin, and 50 µg/mL streptomycin (15140-122, GibcoTM, Thermo Fisher Scientific, Waltham, MA, USA) (DMEM10%) with a 10-mL and a 1-mL pipette. To obtain a single cell suspension, the cells were strained through a 70-µm cell strainer. The suspension was centrifuged at 800×*g* for 5 min. The supernatant was discarded, and the cells were re-suspended in 10 mL DMEM10% pre-heated to 37 °C. The cells were seeded on poly-L-ornithine (PLO)-(P0671, Sigma-Aldrich, St. Louis, MO, USA)-coated flasks and incubated at 37 °C and 5% CO_2_. The flasks were not moved for the next 4 days. Afterward, other glial cell types were eliminated by shaking the flasks at 37 °C for 2 h at 120 rpm and discarding the old medium. The astrocyte culture was passaged two times by application of 0.1% trypsin (15090-046, GibcoTM, Thermo Fisher Scientific, Waltham, MA, USA) in 2% EDTA/PBS (8043.2, Carl Roth GmbH, Karlsruhe, Germany). During the whole experiment, medium was changed every 3 days until the cell layer became confluent. In passage 3, the cells were used for experiments. After the third sub-culturing, the cells were treated with medium containing decreasing concentrations of glucose. We used the following concentrations: 25 mM which is the glucose concentration used in standard, commercial DMEM, 2 mM which is slightly under the physiological glucose concentration of the rat brain (2.4 ± 0.1 mM), expected to induce chronic undernutrition but not massive cell death (Silver and Erecińska 1994) and 0 mM as a positive control. DMEM without glucose (11966025, GibcoTM, Thermo Fisher Scientific, Waltham, MA, USA) was supplemented with 0.5% fetal calf serum, pyruvate (11360-039, GibcoTM, Thermo Fisher Scientific, Waltham, MA, USA), penicillin, and streptomycin. To obtain a 25-mM glucose medium, a corresponding amount of d-glucose (X997.2, Carl Roth GmbH, Karlsruhe, Germany) was added. Then, the medium was filtered sterile. The 2-mM medium was prepared by an appropriate dilution of the 25-mM medium with 0 mM basal medium. The experiments lasted a total of 15 days. Samples were taken every 3 days.

### Cell Viability and Metabolic Activity Assay

To investigate cell viability and metabolic activity, the CytoTox 96® non-radioactive cytotoxicity assay (G1780, Promega, Madison, WI, USA) and the CellTiter-Blue® cell viability assay (G8081, Promega, Madison, WI, USA) were used according to the manufacturer’s instructions. Astrocytes were seeded in a 96-well opaque-walled tissue culture plate (655090, Greiner Bio One International, Kremsmünster, Austria) and treated with different glucose concentrations as previously described. One group of cells was treated with lysis solution to serve as lysis control, whereas the medium without cells served as blank control. Measurements were conducted with a Tecan infinite M200 plate reader and processed with the i-control 1.10 software. Experiments were performed with two biological and eight technical replicates each.

### Immunofluorescence Labeling and Morphological Characterization

**Fig. 1:**
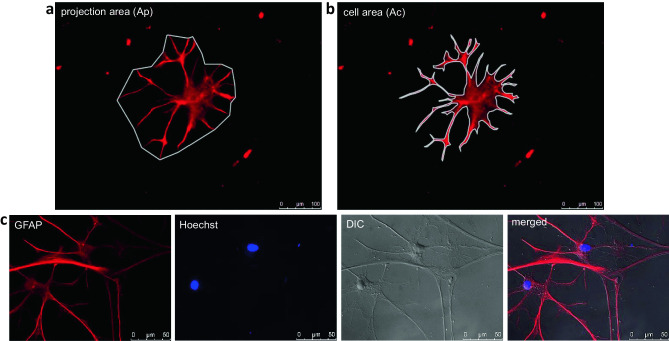
Analysis of astrocyte morphology (*n* = 2). The projection area (Ap) **a** and the cell area (Ac) **b** were measured. Ap is defined as the area that includes the cells’ most prominent processes. Ac describes the area which is actually occupied by a cell. To confirm the morphology, DIC images of GFAP stained cells were taken **c**. GFAP-positive cell area and the area visible through DIC filter match very well

For immunofluorescence labeling, cells were fixed with 3.7% paraformaldehyde (CP10.2, Carl Roth GmbH, Karlsruhe, Germany). For permeabilization of cell membranes, cells were treated with 0.2% Triton X-100 (3051, Carl Roth GmbH, Karlsruhe, Germany) in 1× PBS for 15 min. Then, cells were exposed to blocking buffer (1% BSA and 2% FCS in PBS) to block nonspecific binding sites for 1 h. Cells were incubated overnight at 4 °C with an anti-GFAP antibody diluted in blocking buffer (concentration: 1:1000, ab4674, Abcam, Cambridge, UK). The next day, the secondary antibody conjugated with fluorescent dye Alexa 594 (concentration: 1:500; A-11042, InvitrogenTM, Thermo Fisher Scientific, Carlsbad, CA, USA) was applied. Cell nuclei were counterstained with Hoechst 33342 (H3570, InvitrogenTM, Thermo Fisher Scientific, Carlsbad, CA, USA). Since GFAP staining does not depict the entire cell cytoplasm/surface of an astrocyte, but only the GFAP-positive parts, we additionally used differential interference contrast (DIC) microscopy to confirm the morphology. DIC microscopy is a technique to better visualize specimens with little or no contrast by producing a pseudo 3D effect (Keevil and Walker 1992). The GFAP-positive cell area and the area visible through a DIC filter match very well as shown in Fig. 1c. Fluorescence and DIC images were taken with a Leica DMI 6000 B microscope (Leica Biosystems).

For morphological characterization, a ramification index was calculated (Becker et al. 2018). The ramification index is used to determine the morphological appearance and changes of glia cells (Eder et al. 1999; Heppner et al. 1998). It is based on the assumption that microglia and astrocytes are identified as resting if they have small soma as well as thin and ramified processes. Activated glia cells on the other hand show hypertrophy with retracted processes (Rinaldi et al. 2015). Therefore, the projection area (Ap) (Fig. 1a) and the cell area (Ac) (Fig. 1b) were measured using ImageJ 1.48v (National Institutes of Health, USA). The following formula was used:

$$\frac{Cell \ area}{Projection \ area}= \frac{{A}_{c}}{{A}_{p}}$$

Ap is defined as the area that include the cells’ most prominent processes (Fig. 1a). Resting astrocytes are characterized by small cell bodies and long ramified processes resulting in small cell areas and large projection areas, whereas activated cells show a nearly identical projection and cell area due to hypertrophy of somata and processes (Becker et al. 2018; Rinaldi et al. 2015). Therefore, the ramification index for activated cells converges to 1. Experiments were performed with two biological and three technical replicates each.

### RNA Extraction and Real-Time Quantitative PCR

RNA was extracted using peqGold RNA TriFast according to manufacturer’s instructions (30-2010, VWR, Germany). RNA concentration and purity was measured with a NanoDrop 1000 device (PeqLab, Germany). To synthesize cDNA, the Moloney Murine Leukemia Virus Reverse Transcriptase (M-MLV) RT-kit (28025-021, Thermo Fisher Scientific, Waltham, MA, USA) and random hexanucleotide primer (48190-011, Thermo Fisher Scientific, Waltham, MA, USA) were used. The concentration of total RNA was adjusted to 1 µg/mL. Gene expression was measured using RT-qPCR and the MyIQ detection system (Biorad, Germany). The relative quantification of RNA expression was calculated using the ΔΔCt-method. The relative amount of the target gene was divided by the relative amount of the reference gene CycloA. For miRNA 155-5p, the averaged, relative amounts of 103-3p and 107-3p were used as reference genes. For each day of measuring, the respective 25 mM condition was used as a control and set to 100%. The sequences of the primers used in this study are listed in Table 1.

**Table 1:** List of primers used in the study

Primer	Sequence	Product size (bp)^a^	AT^b^ (°C)
ATF4	5′ GCTCTTCACGAAACCCAGCA 3′ 3′ CCAACACTTCGCTGTTCAGGA 5′	129	62
ATF6	5′ TTCTTCAACTCAGCACGTTCC 3′ 3′ AGGCTTCTCTTCCTTCAGTGG 5′	124	60
Bax	5′ CATCCAGGATCGAGCAGAGAG 3′ 3′ CAATTCGCCTGAGACACTCG 5′	112	65
Bcl2	5′ AAGCCGGGAGAACAGGGTAT 3′ 3′ CGCGGAGTCTTCATCTCCAG 5′	107	65
C3	5′ ACTGGTCAACATGGGGCAGT 3′ 3′ TCGAAACTGGGCAGCACGTA 5′	108	60
CHOP	5′ TGTTGAAGATGAGCGGGTGG 3′ 3′ GCTTTCAGGTGTGGTGGTGT 5′	108	65
Cx43	5′ GGTGTCCTTGGTGTCTCTCG 3′ 3′ CTTCACGCGATCCTTAACGC 5′	79	64
CycloA	5′ GGCAAATGCTGGACCAAACAC 3′ 3′ TTAGAGTTGTCCACAGTCGGAGATG 5′	196	65
IL1β	5′ TGGCAACTGTCCCTGAACTC 3′ 3′ GTCGAGATGCTGCTGTGAGA 5′	170	62
IL6	5′ GGTCTGTTGTGGGTGGTATCC 3′ 3′ CCAGTTGCCTTCTTGGGACT 5′	101	60
NLRP3	5′ TCTGTTCATTGGCTGCGGAT 3′ 3′ GCCTTTTTCGAACTTGCCGT 5′	314	65
Psmb8	5′ CGGGACACTACAGTTTCTCCGT 3′ 3′ GCCGTGCGCCATTTCAATCT 5′	128	64
S100a10	5′ CCCTCTGGCTGTGGACAAAAT 3′ 3′ AATGATGAGCCCCGCCACTA 5′	100	60
Slc2a1	5′ TGGCGGCGGTCCTATAAAAA 3′ 3′AGACCCTGCAACCTAAACCG 5′	92	62
Slc2a2	5′ ACACCAGCACATACGACACCA 3′ 3′AGCCACCCACCAAAGAACGA 5′	137	60
TNFα	5′ GGAGGGAGAACAGCAACTCC 3′ 3′ TCTGCCAGTTCCACATCTCG 5′	168	64
sXBP1	5′ TGCTGAGTCCGCAGCAGGTG 3′ 3′ GCTGGCAGACTCTGGGGAAG 5 ‘	169	60
tXBP1	5′ GAAAGAAAGCCCGGATGAGC 3′ 3′ TCCCCAAGCGTGTCCTTAAC 5′	147	60
103-3p	5′ GCAGAGCAGCATTGTACAG 3′ 3′ GGTCCAGTTTTTTTTTTTTTTTCATAG 5′	–	57
107-3p	5′ GCAGAGCAGCATTGTACAG 3′ 3′ GGTCCAGTTTTTTTTTTTTTTTGATAG 5′	–	56
155-5p	5′ CGCAGTTAATGCTAATTGTGATAG 3′ 3′ AGGTCCAGTTTTTTTTTTTTTTTACC 5′	–	55

^a^bp = base pairs, ^b^AT = annealing temperature

### Protein Isolation, SDS-Page, and Western Blot

Protein was isolated after semi-starvation by homogenizing cells in RIPA buffer which consists of 150 mM NaCl (27810.295, VWR, Germany), 1% (v/v) Nonidet P-40 (74385, Sigma, Igepal, CA), 0.1% SDS (sodium dodecyl sulfate) (4360.2, Carl Roth GmbH, Karlsruhe, Germany), 0.5% sodium deoxycholate (D6750-256, Sigma, Igepal, CA), and 50 mM Tris–HCl (9090.3, Carl Roth GmbH, Karlsruhe, Germany), supplemented with Complete Mini, a protease inhibitor cocktail (11836170001, Roche Diagnostics, Grenzach-Wyhlen, Germany). The pH was adjusted to 8.0. Protein concentration was measured with the PierceTM BCA Protein Assay kit (23225, Thermo Fisher Scientific, Waltham, USA) according to the manufacturer’s instructions. Fifteen micrograms of protein per lane was loaded on a 12% (v/v) discontinuous sodium dodecyl sulfate-polyacrylamide gel and separated by electrophoresis (SDS-PAGE). Afterward, the proteins were transferred to a polyvinylidene difluoride (PVDF) membrane (03010040001, Roche Diagnostics, Grenzach-Wyhlen, Germany). The membranes were blocked with 5% skimmed milk at room temperature and then incubated overnight at 4 °C with an anti-CHOP or anti-ATF4 antibody (concentration: 1:1000, sc-7351/sc-390063, Santa Cruz Biotechnology, Dallas, TX, USA) diluted in milk. After washing the membranes in TBS-T (0.15 M NaCl, 50 mM Tris–HCL, 0.05% Tween 20 (9127.1, Carl Roth GmbH, Karlsruhe, Germany)), they were incubated with horseradish peroxidase–conjugated secondary antibody for 2 h at room temperature. The results were visualized by the enhanced chemiluminescence method (ECL Plus; 32109, Thermo Fisher Scientific, Waltham, MA, USA) and a standard protocol. As a loading control, the membranes were incubated with an antibody against β-actin (1:5000, sc-1616, Santa Cruz Biotechnology, Dallas, TX, USA).

### Data Analysis

In total, eight independent in vitro experiments (independent platings = *n*) were performed with 2 wells each per group unless otherwise stated. The exact number *n* for the respective experiments is given in the legends of the figures. All data are indicated as arithmetic means ± SEM. The Shapiro-Wilk test was performed to test for normal distribution. If normal distribution was not given, data were transformed using Box-Cox with an optimal *λ*. Statistical differences were evaluated by two-way analysis of variance (ANOVA) followed by Holm-Sidak’s post hoc test using GraphPad Prism 8 (GraphPad software). Differences between the groups with *p* ≤ 0.05 are considered statistically significant.

## Results

**Fig. 2:**
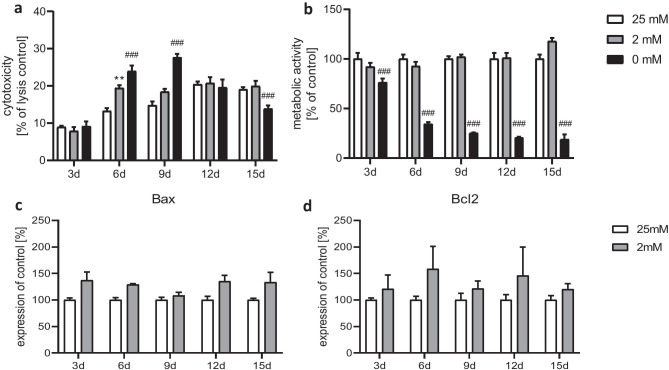
Effect of chronic glucose starvation on cytotoxicity **a** and metabolic activity **b** in cultured primary rat astrocytes. Treatment with medium containing 0 mM glucose increased the LDH release and decreased the metabolic activity. Both parameters were not affected under growing conditions using 2 mM glucose except for a significant increase of cytotoxicity at day 6. Measurements of the gene expression levels of the apoptotic markers Bax **c** and Bcl2 **d** showed no significant differences. Data represents the means ± SEM from two independent experiments (*n* = 2) for the assays and three experiments (*n* = 3) for the RT-qPCR. **p ≤ 0.01 compared to 25 mM, ^###^*p* ≤ 0.001 compared with 25 mM and 2 mM

Treatment of astrocytes with a positive control medium containing 0 mM glucose led to an elevated cytotoxicity on days 6 and 9 in comparison with the 25 mM and 2 mM conditions (Fig. 2a). The metabolic activity was massively reduced from day 3 onward (Fig. 2b). After the administration of medium with 2 mM glucose, cells showed an increased cytotoxicity only on day 6 (Fig. 2a), whereas the general metabolic activity was not significantly affected at any time point (Fig. 2b). We assume that semi-starvation triggers distinct vulnerability of cells which are already hampered from the start of cultivation, and starvation then adds the final droplet. In favor of this idea is the fact that this process comes to a standstill after 9 days. Since dying cells are replaced by, although at this stage, lower mitotic rates, the metabolic activity as a total does not seem to be affected.

Both glucose concentrations (25 mM and 2 mM) did apparently not differ much in their effectiveness with respect to cytotoxicity and metabolic activity (except for cytotoxicity at day 6). Therefore, we used the 25 mM and 2 mM glucose conditions for all further experiments. Gene expression of the apoptotic markers Bax and Bcl2 was slightly but not significantly increased in the semi-starved astrocytes (Fig. 2c, d) confirming the results of the LDH assay.

**Fig. 3:**
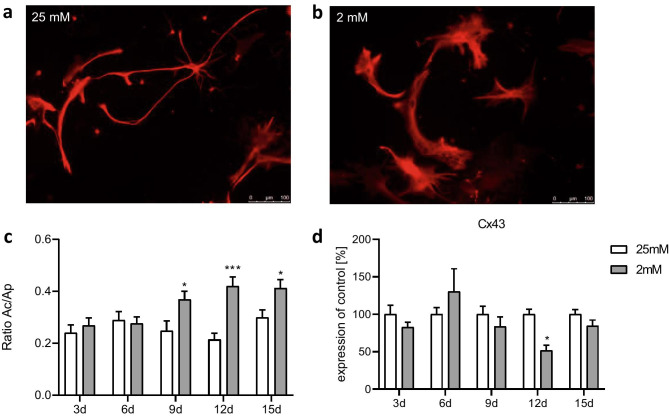
Effect of chronic glucose starvation on cell morphology in cultured primary rat astrocytes indicated by an immunofluorescence staining with GFAP **a**–**c** and a RT-qPCR of connexin 43 **d**. Under chronic glucose starvation, the astrocyte morphology changed from a resting state with small cell bodies and long, slender processes **a** to a reactive state with broad cell bodies and short processes **b**. Under 2 mM glucose conditions, the ratio of the cell area and the projection area converged toward 0.5 from day 9 onward **c**. The expression of the gene encoding connexin 43 was reduced in astrocytes treated with medium containing 2 mM glucose on day 12 **d**. Data represent the means ± SEM from two independent experiments (*n* = 2) for the GFAP staining and three experiments (*n* = 3) for the RT-qPCR. *, **, ***p ≤ 0.05; *p* ≤ 0.01; *p* ≤ 0.001 compared with 25 mM

Glucose starvation affected the morphology of primary astrocytes (Fig. 3). After treatment with medium containing 25 mM glucose for 15 days, most of the astrocytes retained small cell bodies with long and slender processes (Fig. 3a). This phenotype typically defines a resting state (Prah et al. 2019). Astrocytes grown with a medium containing 2 mM glucose showed hypertrophic soma and processes (Fig. 3b). The ramification index calculated by the ratio of the cell area (Ac) divided by the projection area (Ap) was significantly increased from day 9 onwards under 2 mM glucose conditions (Fig. 3c). Expression of the gene of connexin 43 (Cx43), an integral part of gap junctions (Nagy and Rash 2000), tended to be reduced from day 9 on, reaching significance on day 12 in cells exposed to the 2 mM glucose medium compared with the 25 mM condition (Fig. 3d).

**Fig. 4:**
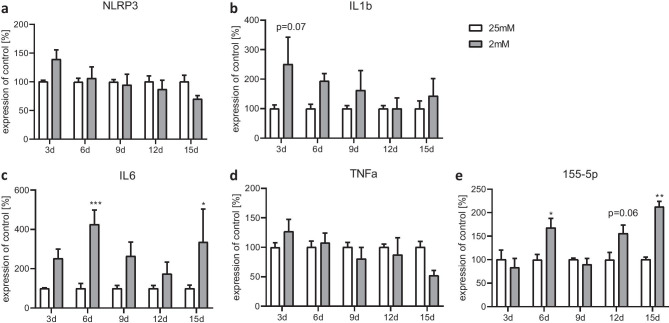
Effect of chronic glucose starvation on expression levels of genes associated with inflammation in cultured primary rat astrocytes. Under chronic glucose starvation, NLRP3 **a** and IL1β **b** gene expression showed a trend to be elevated on day 3 under 2 mM glucose conditions. Chronic glucose starvation doubled the gene expression of IL6 on days 3, 6, 9, and 15, becoming significant on days 6 and 15 **c** in the 2 mM glucose conditions. The expression of TNFα was not statistically influenced at any time point **d**. The expression of mi-RNA 155-5p was significantly increased at days 6 and 15 **e**. Data represent the means ± SEM from three independent experiments (*n* = 3). *, **, ***p ≤ 0.05; *p* ≤ 0.01; *p* ≤ 0.001 compared with 25 mM

In the next set of experiments, we analyzed the expression levels of genes associated with inflammation including NOD-, LRR- and pyrin domain-containing protein 3 (NLRP3); interleukin 1β (IL1β); interleukin 6 (IL6); tumor necrosis factor alpha (TNFα); and the microRNA (mi-RNA) 155-5p. NLRP3 and IL1b were high at the beginning of culturing under both conditions but then stepwise decreased (Fig. 4a, b). NLRP3 and IL1β were not differentially affected under 2 mM glucose conditions compared with 25 mM glucose. The pro-inflammatory gene IL6 was significantly elevated at days 6 and 15 and showed a trend to an induction on days 3, 9, and 12 under 2 mM glucose conditions (Fig. 4c), whereas the expression of TNFα was not changed (Fig. 4d). The mi-RNA 155-5p expression was increased on days 6 and 15 (Fig. 4e).

**Fig. 5:**
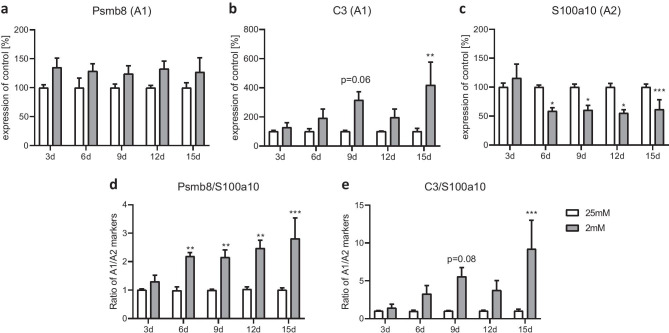
Effect of chronic glucose starvation on the expression levels of genes related to the A1 and A2 phenotype in cultured primary rat astrocytes. Under chronic glucose starvation, the gene expressions of A1 markers Psmb8 **a** and C3 **b** showed increases that were significant for C3 at day 15, whereas the gene expression of the A2 marker S100a10 was significantly decreased **c** in astrocytes treated with 2 mM glucose from day 6 onward. The ratios of these genes showed a clear shift to a A1-like phenotype in the semi-starved astrocytes **d**, **e**. Data represents the means ± SEM from three independent experiments (*n* = 3). *, **, ***p ≤ 0.05; *p* ≤ 0.01; *p* ≤ 0.001 compared with 25 mM

We further studied genes related to the different astrocytic phenotypes, i.e., A1 and A2, under the different glucose conditions. The pro-inflammatory A1 marker C3 was significantly higher expressed in semi-starved astrocytes on day 15, whereas the gene expression of Psmb8 was nominally but not significantly increased at all studied time points (Fig. 5a, b). S100a10, a typical anti-inflammatory A2 marker, was significantly reduced from day 6 onward under 2 mM conditions (Fig. 5c). A1/A2 ratios of those genes showed a clear shift toward the A1 phenotype under semi-starvation conditions (Fig. 5d, e).

**Fig. 6:**
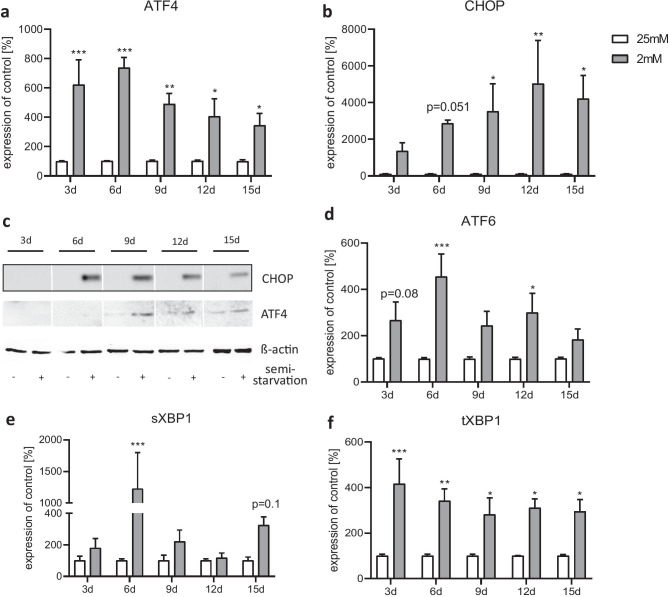
Effect of chronic glucose starvation on the expression levels of genes related to the unfolded protein response (UPR) in cultured primary rat astrocytes. Chronic glucose starvation significantly increased gene expression rates of ATF4 **a**, CHOP **b**, ATF6 **d** and XBP1 in its spliced version (sXBP1) **e** as well as the amount of total XBP1 (tXBP1) **f** in cells grown with 2 mM glucose. At the protein level, CHOP was measureable from day 6 and ATF4 from day 9 onward in the semi-starved cells **c**. Data shows the means ± SEM from three independent experiments (*n* = 3) for the gene expression and representative results for the protein level. *, **, ***p ≤ 0.05; *p* ≤ 0.01; *p* ≤ 0.001 compared with 25 mM

We investigated the cellular UPR response under semi-starvation by studying the expression of the UPR-associated genes. Gene expression of ATF4 was significantly increased at all measured time points under 2 mM glucose conditions (Fig. 6a). The induced C/EBP homologous protein (CHOP) showed significantly increased gene expression from day 9 onward under semi-starvation (Fig. 6b). At the protein level, CHOP was detectable after 6 days and ATF4 after 9 days of semi-starvation (Fig. 6c). ATF6 was significantly elevated on days 6 and 12 (Fig. 6d). The other days in culture, we found a tendency toward higher expression levels under 2 mM conditions. The gene encoding the spliced version of X-box binding protein 1 (sXBP1) revealed a significant induction on day 6 and a tendency toward an induction on day 15 (Fig. 6e). The total amount of XBP1 (tXBP1) was increased at all measured time points in semi-starved astrocytes (Fig. 6f).

**Fig. 7:**
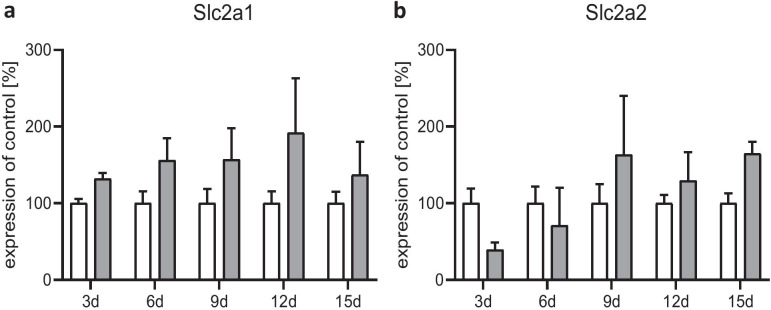
Effect of chronic glucose starvation on the expression levels of glucose transporters in cultured primary rat astrocytes. Glucose semi-starvation did not significantly alter the gene expression of GLUT1 (gene: Slc2a1) and GLUT2 (gene: Slc2a2). Data represents the means ± SEM from three independent experiments (*n* = 3)

In a last step, we measured the gene expression of the glucose transporters GLUT1 (gene: Slc2a1) and GLUT2 (gene: Slc2a2). The expression of both mRNAs showed a slight but not significant increase under the 2 mM condition (Fig. 7a, b). Slc2a1 was stepwise induced (Fig. 7a), whereas Slc2a2 initially declined and then returned to basal level afterward from day 6 onwards (Fig. 7b).

## Discussion

In the present study, we investigated the long-term effect of reduced glucose concentration on astrocytes to better understand the alterations and functions of astrocytes in the brain under food deprivation as seen in eating disorders like AN. Consistent with previous studies in patients suffering from AN and the ABA animal model, we could demonstrate an effect of long-term semi-starvation on brain cells. Astrocytes react to glucose deprivation with an inflammatory response. This response is characterized by elevated pro-inflammatory cytokines and genes associated with the inflammasome. Furthermore, a shift toward the pro-inflammatory A1-like phenotype and an altered morphology indicate an increased reactivity. An elevated UPR points toward further stress responses associated with semi-starvation. These findings further extend our understanding of the underlying cellular pathophysiology of semi-starvation and point to a role of inflammatory processes in the brain.

In our in vitro model, we reduced the glucose concentration in an astrocyte culture to 2 mM instead of 25 mM over 15 days. It has already been shown that a reduction of the glucose concentration leads to a hunger situation in astrocytes. Astroglia can store small amounts of glycogen which they can metabolize in case of a deficiency of glucose (Kreft et al. 2012). Using an astrocyte in vitro model, a significantly reduced glycogen storage has been demonstrated after 60 min without glucose (Dringen et al. 1993; Dringen and Hamprecht 1993). Even after culturing with reduced glucose concentrations of 2 mM, rat astrocytes show a significantly reduced amount of glycogen compared with astrocytes supplied with 22 mM glucose medium (Abe et al. 2006).

In our study, an increased ramification index indicates morphological changes and an increased reactivity of astrocytes under semi-starvation. Similar alterations were found in the hippocampus of young female rats in the dehydration-induced anorexia model (Reyes-Haro et al. 2016). The increased expressions of vimentin and nestin in the hippocampus of these animals also point to an elevated number of reactive astrocytes (Reyes-Haro et al. 2016).

Gene expression of Cx43 was decreased on day 12 in the semi-starved astrocytes. Cx43 is the main component of gap junctions in astrocytes in the CNS (Nagy and Rash 2000) and enables the transport of glucose and other small compounds within the astrocytic network (Allard et al. 2014). A decreased expression of this protein would clearly influence glucose and lactate distribution and could possibly limit the supply of neurons (Clasadonte et al. 2017). Therefore, we searched for a functional parameter which might follow the observed morphological changes (“function follows form”). However, there was no clear-cut evidence. Nevertheless, we think this information is important, since it shows that the astrocyte network despite low nutrition seems to be intact in vitro.

In the same context, the expression of glucose transporters was examined. No significant differences in gene expression levels were found for the two glucose transporters GLU1 and GLU2 which are expressed on astrocytes (Arluison et al. 2004; Maher et al. 1994). However, a gradual induction can be observed in the course of the experiment. Increased expression of glucose transporters occurs with an undersupply of glucose as a compensatory mechanism (Koepsell 2020). Such a response could also take place in our semi-starvation model and might be the underlying mechanism behind the slowly increasing expression of glucose transporters during culturing.

Our results showed an increased expression of pro-inflammatory cytokines and genes associated with the inflammasome. IL1β and NLRP3 gene expression levels are initially high and then decrease in the course of culturing in the 2-mM condition and become stable at a certain level. This could reflect the initial handling of cells to bring them into culture. This particularly means that astroglia at the beginning of culturing become somehow activated since a number of cells die during the first days and release toxic compounds such as damage-associated molecular patterns (DAMPs) which in turn activate the inflammasome system of astrocytes (Vénéreau et al. 2015). This lasts for a few days and then comes to a homeostasis. Importantly, the starvation does not further boost or prolong this phenomenon. In addition, the expression of mi-RNA 155-5p was significantly increased after 12 days. In general, mi-RNA can affect more than one target gene/mRNA (Cai et al. 2009). mi-RNA 155-5p is particularly interesting in our starvation model, since it is involved in the regulation of glucose metabolism (Lin et al. 2016). Furthermore, mi-RNA 155-5p expression is decreased in the blood of diabetes mellitus patients and in a mouse model of obesity (Gaudet et al. 2016; Polina et al. 2019). In vitro, the expression of mi-RNA 155-5p was decreased after 48 h serum starvation in human peripheral blood mononuclear cells (Rahmani et al. 2018). In addition, the mi-RNA 155-5p is known to be involved in the regulation of the inflammasomes. It indirectly affects NLRP3 expression by inhibiting the adapter protein MyD88 (Boxberger et al. 2019), which is needed for priming of NLRP3 (Kalliolias and Ivashkiv 2016; Latz et al. 2013). In this way, elevated mi-RNA 155-5p can inhibit MyD88, prevent the formation of an inflammasome, and counteract a pro-inflammatory reaction. At the same time, the pro-inflammatory cytokine IL6 was significantly induced on day 15. The differences in the expression pattern of the cytokines could be explained by different induction pathways. IL1β is mainly activated via pathogen-associated molecular patterns (PAMPs) and in combination with inflammasomes (Lopez-Castejon and Brough 2011). IL6 on the other hand is additionally activated by DAMPs and other cytokines like TNFα (Tanaka et al. 2014). One of these other activation pathways could explain the induction of IL6 expression on days 6 and15, while the expression of NLRP3 and IL1β remains suppressed probably due to the inhibition by mi-RNA 155-5p. Increased pro-inflammatory cytokine expression fits to findings in patients suffering from AN. These studies showed an elevated expression of IL6, IL1β, and TNFα in the blood of patients with AN (Dalton et al. 2018; Solmi et al. 2015). Similar increases of IL6 and IL1β gene expressions were found in the hippocampus of young female rats in the dehydration-induced anorexia model (Ragu-Varman et al. 2019). Astrocytes in vitro showed cell impairment and death due to long-term exposure to IL1β and TNFα (van Kralingen et al. 2013). A mild but chronic neuroinflammation occurring during semi-starvation possibly contributes to the astrocyte loss measured in cortex and corpus callosum of female rats in a starvation model (Frintrop et al. 2018).

Models of other diseases already showed that increased inflammation may be important in development and progression of diseases. The inhibition of MyD88 by mi-RNA 155-5p has been shown to alleviate depressive-like behavior in rats (Jia et al. 2018), a common comorbidity of AN (Marucci et al. 2018). It is known that pro-inflammatory cytokines are sufficient to induce symptoms of depression in patients and that IL1β as well as IL6 play a prominent role in this mechanism (Maes et al. 2009).

In our study, we could demonstrate a significantly increased expression of CHOP, a gene associated with the UPR. An elevated level of CHOP expression was found in an animal model of depression (Timberlake et al. 2018). Since CHOP and related UPR are able to induce the expression of IL1β and can cause inflammatory reactions, a connection between the increased UPR and the inflammatory reaction in depression seems possible (Endo et al. 2006; Kim et al. 2008). In addition to depression, impaired learning has been demonstrated in patients with AN as well as the ABA animal model (Green et al. 1996; Paulukat et al. 2016). Increased UPR, as we found in this study, has already been shown to be associated with impaired learning (Moreno et al. 2012). Inhibition of ATF4 expression, which is significantly induced in semi-starved astrocytes in our model, increases learning and memory abilities in wild-type mice (Sidrauski et al. 2013). Furthermore, ATF4 prevents expression of genes mediated by cAMP response element-binding protein (CREB) which is probably necessary for long-term memory formation, synaptic plasticity, and behavioral learning (Chen et al. 2003). One of the proteins mediated by CREB is the brain-derived neurotrophic factor (BDNF) (Esvald et al. 2020) which was found to be decreased in patients suffering from AN (Brandys et al. 2011). In an in vitro model of astrocytes, it was demonstrated that an over-activation of the UPR pathway including ATF4 and CHOP generates a reactive state in astrocytes that alters the composition of proteins expressed by these cells, resulting in loss of function in synapses (Smith et al. 2020).

Cell culture models have previously been used to study diseases associated with aberrant nutrient intake, e.g., diabetes, and obesity. Diabetes is a disease associated with phases of severe hypoglycemia (Yale et al. 2018). In an in vitro model of diabetes, short but severe glucose deprivation leads to death of neurons. An inhibition of the pathway expressing ATF4 and CHOP prevents this neuronal death (de la Cadena et al. 2014). Excessive activation of UPR can cause cell death by producing apoptotic proteins (Hetz et al. 2006). This might explain why inhibition of UPR avoids neuronal damage. In astrocyte cell cultures, the release of apoptotic proteins is caused by inflammation (Guthrie et al. 2016). In another in vitro study of diabetes, recurrent hypoglycemic phases lead to a shift from glucose metabolism to fatty acid oxidation in human primary astrocytes (Weightman Potter et al. 2019). The same effect was shown after prolonged inflammation induced by LPS stimulation in astrocytes in vitro (Robb et al. 2020). In addition, XBP1 regulates components of the glucose metabolism and lipogenesis. Therefore, a comparable influence is attributed to this protein (Piperi et al. 2016). Since we found an inflammatory reaction as well as an increased expression of tXBP1 and a time-specific induction of the sXBP1, a similar effect might occur during the long-term semi-starvation in our experiments.

In contrast to AN or diabetes, obesity is characterized by an oversupply of nutrients. Nevertheless, increased inflammation and UPR in astrocytes, as found in our study of semi-starvation, are also important in this form of malnutrition. Treatment with long-chain fatty acids induces an inflammatory response in an in vitro astrocyte model of obesity (Gupta et al. 2012). In the blood of patients with obesity, high levels of free fatty acids provoke ER stress which induces low but chronic inflammation (Boden 2009). In animal models of obesity and type 2 diabetes, hypothalamic astrocytes undergo structural changes in the form of synapses loss of neurons (Horvath et al. 2010) which are associated with chronic hypothalamus inflammation due to high-fat diet (Cai and Liu 2011).

The hypothalamus is an important structure related to the physiological regulation of various metabolic processes as well as implicated in the development and dysregulation in disease. Hypothalamic astrocytes are crucial for sensing nutrients and hormones (García-Cáceres et al. 2016; Kim et al. 2014). Astrocytes express receptors for ghrelin and leptin (Frago and Chowen 2017; Kim et al. 2014) which play an important role in the development of hunger and the feeling of satiety (Yeung and Tadi 2020). In obesity, it is presumed that neuroinflammation triggered by astrocytes attenuates the leptin signal, leading to an increased feeling of hunger and subsequently weight gain (García-Cáceres et al. 2013) despite the fact that blood leptin levels of obesity patients are high (Considine et al. 1996). A similar effect could also occur in patients with AN who show increased blood ghrelin levels (Otto et al. 2001). Since ghrelin receptors are expressed by hypothalamic astrocytes (Frago and Chowen 2017), an inflammatory reaction triggered by these cells could possibly influence the ghrelin sensing in underweighted patients. This assumption is supported by the presence of increased inflammatory parameters in patients with AN (Dalton et al. 2018; Solmi et al. 2015) and an increased cytokine expression as demonstrated in our model of semi-starved astrocytes. For all these reasons, metabolic inflammation and the concomitant increased UPR have been associated with the progression and severity of metabolic diseases.

## Conclusion

There is good evidence that an aberrant intake of nutrients causes cellular stress reactions. It seems to be irrelevant whether the trigger is undernutrition as in our case or overnutrition as in obesity. Astrogliosis and increased UPR has been demonstrated previously to play an important role in the metabolic diseases diabetes and obesity as well as in depression. Here, we showed morphological and genetic evidence for pro-inflammatory astrocyte subtype-induction and increased UPR responses indicating that inflammatory processes are a relevant factor in undernutrition. How these inflammatory processes might be involved in astrocyte reduction found in the ABA model of AN needs to be evaluated in further experiments.

## Data Availability

The datasets generated and analyzed during the current study are available from the corresponding author on reasonable request.

## References

[CR1] Abe T, Takahashi S, Suzuki N (2006). Oxidative metabolism in cultured rat astroglia: effects of reducing the glucose concentration in the culture medium and of D-aspartate or potassium stimulation. J Cereb Blood Flow Metab.

[CR2] Allard C (2014). Hypothalamic astroglial connexins are required for brain glucose sensing-induced insulin secretion. J Cereb Blood Flow Metab.

[CR3] Arluison M, Quignon M, Nguyen P, Thorens B, Leloup C, Penicaud L (2004). Distribution and anatomical localization of the glucose transporter 2 (GLUT2) in the adult rat brain–an immunohistochemical study. J Chem Neuroanat.

[CR4] Association AP (2013). Diagnostic and statistical manual of mental disorders: DSM-5.

[CR5] Becker B (2018). Effect of intrastriatal 6-OHDA lesions on extrastriatal brain structures in the mouse. Mol Neurobiol.

[CR6] Boden G (2009). Endoplasmic reticulum stress: another link between obesity and insulin resistance/inflammation?. Diabetes.

[CR7] Boxberger N, Hecker M, Zettl UK (2019). Dysregulation of inflammasome priming and activation by microRNAs in human immune-mediated diseases. J Immunol.

[CR8] Brandys MK, Kas MJ, van Elburg AA, Campbell IC, Adan RA (2011). A meta-analysis of circulating BDNF concentrations in anorexia nervosa. The world journal of biological psychiatry : the official journal of the World Federation of Societies of Biological Psychiatry.

[CR9] Bruch H (1993) The Fröhlich syndrome: report of the original case. 1939 Obes Res 1:329–331. 10.1002/j.1550-8528.1993.tb00628.x 10.1002/j.1550-8528.1993.tb00628.x 16350582

[CR10] Buehren K, Konrad K, Schaefer K, Kratzsch J, Kahraman-Lanzerath B, Lente C, Herpertz-Dahlmann B (2011). Association between neuroendocrinological parameters and learning and memory functions in adolescent anorexia nervosa before and after weight recovery. J Neural Transm.

[CR11] Cai D, Liu T (2011). Hypothalamic inflammation: a double-edged sword to nutritional diseases. Ann N Y Acad Sci.

[CR12] Cai Y, Yu X, Hu S, Yu J (2009). A brief review on the mechanisms of miRNA regulation genomics. Proteomics & Bioinformatics.

[CR13] Castro-Fornieles J (2010). A cross-sectional and follow-up functional MRI study with a working memory task in adolescent anorexia nervosa. Neuropsychologia.

[CR14] Chen A et al (2003) Inducible enhancement of memory storage and synaptic plasticity in transgenic mice expressing an inhibitor of ATF4 (CREB-2) and C/EBP proteins. Neuron 39:655–669. 10.1016/s0896-6273(03)00501-4 10.1016/s0896-6273(03)00501-4 12925279

[CR15] Chen Y, Swanson RA (2003). Astrocytes and brain injury. J Cereb Blood Flow Metab.

[CR16] Clasadonte J, Scemes E, Wang Z, Boison D, Haydon PG (2017) Connexin 43- mediated astroglial metabolic networks contribute to the regulation of the sleep-wake cycle. Neuron 95:1365-1380.e1365. 10.1016/j.neuron.2017.08.022 10.1016/j.neuron.2017.08.022 PMC5617118 28867552

[CR17] Considine RV (1996). Serum immunoreactive-leptin concentrations in normal-weight and obese humans. N Engl J Med.

[CR18] Dalton B, Campbell IC, Chung R, Breen G, Schmidt U, Himmerich H (2018) Inflammatory markers in anorexia nervosa: an exploratory study. Nutrients 10. 10.3390/nu10111573 10.3390/nu10111573 PMC6266841 30355978

[CR19] de la Cadena SG, Hernandez-Fonseca K, Camacho-Arroyo I, Massieu L (2014). Glucose deprivation induces reticulum stress by the PERK pathway and caspase-7- and calpain-mediated caspase-12 activation. Apoptosis.

[CR20] Dringen R, Gebhardt R, Hamprecht B (1993). Glycogen in astrocytes: possible function as lactate supply for neighboring cells. Brain Res.

[CR21] Dringen R, Hamprecht B (1993). Differences in glycogen metabolism in astroglia-rich primary cultures and sorbitol-selected astroglial cultures derived from mouse brain. Glia.

[CR22] Eder C, Schilling T, Heinemann U, Haas D, Hailer N, Nitsch R (1999). Morphological, immunophenotypical and electrophysiological properties of resting microglia in vitro. Eur J Neurosci.

[CR23] Endo M, Mori M, Akira S, Gotoh T (2006). C/EBP homologous protein (CHOP) is crucial for the induction of caspase-11 and the pathogenesis of lipopolysaccharide-induced inflammation. J Immunol.

[CR24] Esvald E-E, Tuvikene J, Sirp A, Patil S, Bramham CR, Timmusk T (2020). CREB family transcription factors are major mediators of BDNF transcriptional autoregulation in cortical neurons. J Neurosci.

[CR25] Falkowska A, Gutowska I, Goschorska M, Nowacki P, Chlubek D, Baranowska-Bosiacka I (2015). Energy Metabolism of the Brain Including the cooperation between astrocytes and neurons, especially in the context of glycogen metabolism. Int J Mol Sci.

[CR26] Feng D, Wei J, Gupta S, McGrath BC, Cavener DR (2009). Acute ablation of PERK results in ER dysfunctions followed by reduced insulin secretion and cell proliferation. BMC Cell Biol.

[CR27] Frago LM, Chowen JA (2017). Involvement of astrocytes in mediating the central effects of ghrelin. Int J Mol Sci.

[CR28] Frintrop L (2018). Reduced astrocyte density underlying brain volume reduction in activity-based anorexia rats. The world journal of biological psychiatry : the official journal of the World Federation of Societies of Biological Psychiatry.

[CR29] Frintrop L (2019). The reduction of astrocytes and brain volume loss in anorexia nervosa—the impact of starvation and refeeding in a rodent model. Transcult Psychiatry.

[CR30] García-Cáceres C (2016). Astrocytic insulin signaling couples brain glucose uptake with nutrient availability. Cell.

[CR31] García-Cáceres C (2019). Role of astrocytes, microglia, and tanycytes in brain control of systemic metabolism. Nat Neurosci.

[CR32] García-Cáceres C, Yi CX, Tschöp MH (2013). Hypothalamic astrocytes in obesity. Endocrinol Metab Clin North Am.

[CR33] Gaudet AD (2016). miR-155 deletion in female mice prevents diet-induced obesity. Sci Rep.

[CR34] Green MW, Elliman NA, Wakeling A, Rogers PJ (1996). Cognitive functioning, weight change and therapy in anorexia nervosa. J Psychiatr Res.

[CR35] Gupta S, Knight AG, Gupta S, Keller JN, Bruce-Keller AJ (2012). Saturated long-chain fatty acids activate inflammatory signaling in astrocytes. J Neurochem.

[CR36] Guthrie LN (2016). Attenuation of PKR-like ER kinase (PERK) signaling selectively controls endoplasmic reticulum stress-induced inflammation without compromising immunological responses. J Biol Chem.

[CR37] Hara M, Matsuda Y, Okumura N, Hirai K, Nakagawa H (1989). Effect of glucose starvation on glucose transport in neuronal cells in primary culture from rat brain. J Neurochem.

[CR38] Heppner FL, Roth K, Nitsch R, Hailer NP (1998). Vitamin E induces ramification and downregulation of adhesion molecules in cultured microglial cells. Glia.

[CR39] Herpertz-Dahlmann B (2015). Adolescent eating disorders: update on definitions, symptomatology, epidemiology, and comorbidity. Child Adolesc Psychiatr Clin N Am.

[CR40] Hetz C (2012). The unfolded protein response: controlling cell fate decisions under ER stress and beyond. Nat Rev Mol Cell Biol.

[CR41] Hetz C (2006). Proapoptotic BAX and BAK modulate the unfolded protein response by a direct interaction with IRE1alpha. Science (New York, NY).

[CR42] Horvath TL (2010). Synaptic input organization of the melanocortin system predicts diet-induced hypothalamic reactive gliosis and obesity. Proc Natl Acad Sci USA.

[CR43] Jia K-K (2018). Chaihu-shugan san inhibits inflammatory response to improve insulin signaling in liver and prefrontal cortex of CUMS rats with glucose intolerance. Biomed Pharmacother.

[CR44] Kacem K, Lacombe P, Seylaz J, Bonvento G (1998). Structural organization of the perivascular astrocyte endfeet and their relationship with the endothelial glucose transporter: a confocal microscopy study. Glia.

[CR45] Kalliolias GD, Ivashkiv LB (2016). TNF biology, pathogenic mechanisms and emerging therapeutic strategies. Nat Rev Rheumatol.

[CR46] Kasischke KA, Vishwasrao HD, Fisher PJ, Zipfel WR, Webb WW (2004) Neural activity triggers neuronal oxidative metabolism followed by astrocytic glycolysis. Science (New York, NY) 305:99–103. 10.1126/science.1096485 10.1126/science.1096485 15232110

[CR47] Keevil C, Walker J (1992). Nomarski DIC microscopy and image analysis. Binary.

[CR48] Kim JG (2014). Leptin signaling in astrocytes regulates hypothalamic neuronal circuits and feeding. Nat Neurosci.

[CR49] Kim I, Xu W, Reed JC (2008). Cell death and endoplasmic reticulum stress: disease relevance and therapeutic opportunities. Nat Rev Drug Discovery.

[CR50] Koepsell H (2020). Glucose transporters in brain in health and disease. Pflugers Arch.

[CR51] Kreft M, Bak LK, Waagepetersen HS, Schousboe A (2012) Aspects of astrocyte energy metabolism, amino acid neurotransmitter homoeostasis and metabolic compartmentation. ASN Neuro 4:AN20120007. 10.1042/AN20120007 10.1042/AN20120007 PMC3338196 22435484

[CR52] Latz E, Xiao TS, Stutz A (2013). Activation and regulation of the inflammasomes. Nat Rev Immunol.

[CR53] Lee CY, Dallérac G, Ezan P, Anderova M, Rouach N (2016). Glucose tightly controls morphological and functional properties of astrocytes Front Aging Neurosci.

[CR54] Lee JE, Oney M, Frizzell K, Phadnis N, Hollien J (2015) Drosophila melanogaster activating transcription factor 4 regulates glycolysis during endoplasmic reticulum stress G3 (Bethesda, Md) 5:667–675. 10.1534/g3.115.017269 10.1534/g3.115.017269 PMC4390581 25681259

[CR55] Liddelow SA, Barres BA (2017). Reactive astrocytes: production, function, and therapeutic potential. Immunity.

[CR56] Liddelow SA (2017). Neurotoxic reactive astrocytes are induced by activated microglia. Nature.

[CR57] Lin X (2016). MiR-155 enhances insulin sensitivity by coordinated regulation of multiple genes in mice. PLoS Genetics.

[CR58] Lopez-Castejon G, Brough D (2011). Understanding the mechanism of IL-1β secretion. Cytokine Growth Factor Rev.

[CR59] Maes M (2009). The inflammatory & neurodegenerative (I&ND) hypothesis of depression: leads for future research and new drug developments in depression. Metab Brain Dis.

[CR60] Maher F, Vannucci SJ, Simpson IA (1994). Glucose transporter proteins in brain Faseb j.

[CR61] Marucci S, Ragione LD, De Iaco G, Mococci T, Vicini M, Guastamacchia E, Triggiani V (2018). Anorexia nervosa and comorbid psychopathology. Endocr Metab Immune Disord Drug Targets.

[CR62] Mattingly D, Bhanji S (1995). Hypoglycaemia and anorexia nervosa. J R Soc Med.

[CR63] Mergenthaler P, Lindauer U, Dienel GA, Meisel A (2013). Sugar for the brain: the role of glucose in physiological and pathological brain function. Trends Neurosci.

[CR64] Moreno JA (2012). Sustained translational repression by eIF2α-P mediates prion neurodegeneration. Nature.

[CR65] Myer DJ, Gurkoff GG, Lee SM, Hovda DA, Sofroniew MV (2006). Essential protective roles of reactive astrocytes in traumatic brain injury. Brain : a journal of neurology.

[CR66] Nagy JI, Rash JE (2000). Connexins and gap junctions of astrocytes and oligodendrocytes in the CNS Brain research. Brain Res Rev.

[CR67] Otto B (2001). Weight gain decreases elevated plasma ghrelin concentrations of patients with anorexia nervosa. Eur J Endocrinol.

[CR68] Paulukat L (2016). Memory impairment is associated with the loss of regular oestrous cycle and plasma oestradiol levels in an activity-based anorexia animal model. The world journal of biological psychiatry : the official journal of the World Federation of Societies of Biological Psychiatry.

[CR69] Pauwels PJ, Opperdoes FR, Trouet A (1985). Effects of antimycin, glucose deprivation, and serum on cultures of neurons, astrocytes, and neuroblastoma cells. J Neurochem.

[CR70] Pellerin L, Magistretti PJ (1994). Glutamate uptake into astrocytes stimulates aerobic glycolysis: a mechanism coupling neuronal activity to glucose utilization. Proc Natl Acad Sci USA.

[CR71] Piperi C, Adamopoulos C, Papavassiliou AG (2016). XBP1: a pivotal transcriptional regulator of glucose and lipid metabolism. Trends in Endocrinology and Metabolism: TEM.

[CR72] Polina ER, Oliveira FM, Sbruzzi RC, Crispim D, Canani LH, Santos KG (2019). Gene polymorphism and plasma levels of miR-155 in diabetic retinopathy. Endocr Connect.

[CR73] Prah J, Winters A, Chaudhari K, Hersh J, Liu R, Yang S-H (2019). A novel serum free primary astrocyte culture method that mimic quiescent astrocyte phenotype. J Neurosci Methods.

[CR74] Ragu-Varman D, Macedo-Mendoza M, Labrada-Moncada FE, Reyes-Ortega P, Morales T, Martínez-Torres A, Reyes-Haro D (2019). Anorexia increases microglial density and cytokine expression in the hippocampus of young female rats. Behav Brain Res.

[CR75] Rahmani M, Mohammadnia-Afrouzi M, Nouri HR, Fattahi S, Akhavan-Niaki H, Mostafazadeh A (2018). Human PBMCs fight or flight response to starvation stress: increased T-reg, FOXP3, and TGF-β1 with decreased miR-21 and Constant miR-181c levels. Biomed Pharmacother.

[CR76] Reyes-Haro D, Labrada-Moncada FE, Varman DR, Krüger J, Morales T, Miledi R, Martínez-Torres A (2016) Anorexia reduces GFAP+ cell density in the rat hippocampus Neural Plast 2016:2426413–2426413. 10.1155/2016/2426413 10.1155/2016/2426413 PMC4992534 27579183

[CR77] Rinaldi B (2015). Effect of prolonged moderate exercise on the changes of nonneuronal cells in early myocardial infarction. Neural Plast.

[CR78] Robb JL, Hammad NA, Weightman Potter PG, Chilton JK, Beall C, Ellacott KLJ (2020). The metabolic response to inflammation in astrocytes is regulated by nuclear factor-kappa B signaling. Glia.

[CR79] Seitz J, Bühren K, von Polier GG, Heussen N, Herpertz-Dahlmann B, Konrad K (2014) Morphological changes in the brain of acutely ill and weight-recovered patients with anorexia nervosa. A meta-analysis and qualitative review Z Kinder Jugendpsychiatr Psychother 42:7–17; quiz 17–18. 10.1024/1422-4917/a000265 10.1024/1422-4917/a000265 24365959

[CR80] Seitz J, Herpertz-Dahlmann B, Konrad K (2016). Brain morphological changes in adolescent and adult patients with anorexia nervosa. J Neural Transm (Vienna).

[CR81] Sidrauski C (2013). Pharmacological brake-release of mRNA translation enhances cognitive memory. Elife.

[CR82] Silver IA, Erecińska M (1994). Extracellular glucose concentration in mammalian brain: continuous monitoring of changes during increased neuronal activity and upon limitation in oxygen supply in normo-, hypo-, and hyperglycemic animals. J Neurosci.

[CR83] Smith HL (2020). Astrocyte unfolded protein response induces a specific reactivity state that causes non-cell-autonomous neuronal degeneration. Neuron.

[CR84] Sofroniew MV (2014). Astrogliosis. Cold Spring Harb Perspect Biol.

[CR85] Sofroniew MV, Vinters HV (2010). Astrocytes: biology and pathology. Acta Neuropathol.

[CR86] Solmi M, Veronese N, Favaro A, Santonastaso P, Manzato E, Sergi G, Correll CU (2015). Inflammatory cytokines and anorexia nervosa: a meta-analysis of cross-sectional and longitudinal studies. Psychoneuroendocrinology.

[CR87] Tanaka T, Narazaki M, Kishimoto T (2014). IL-6 in inflammation, immunity, and disease. Cold Spring Harb Perspect Biol.

[CR88] Thaler JP (2012). Obesity is associated with hypothalamic injury in rodents and humans. J Clin Investig.

[CR89] Timberlake M, Prall K, Roy B, Dwivedi Y (2018). Unfolded protein response and associated alterations in toll-like receptor expression and interaction in the hippocampus of restraint rats. Psychoneuroendocrinology.

[CR90] van Kralingen C, Kho DT, Costa J, Angel CE, Graham ES (2013). Exposure to inflammatory cytokines IL-1β and TNFα induces compromise and death of astrocytes; implications for chronic neuroinflammation. PLoS One.

[CR91] Vénéreau E, Ceriotti C, Bianchi ME (2015). DAMPs from cell death to new life. Front Immunol.

[CR92] Weightman Potter PG (2019). Basal fatty acid oxidation increases after recurrent low glucose in human primary astrocytes. Diabetologia.

[CR93] Yale J-F, Paty B, Senior PA (2018). Hypoglycemia. Can J Diabetes.

[CR94] Yang J (2017). The multiple roleS of XBP1 in regulation of glucose and lipid metabolism. Curr Protein Pept Sci.

[CR95] Yeung AY, Tadi P (2020) Physiology, obesity neurohormonal appetite and satiety control. In: StatPearls. StatPearls Publishing, StatPearls Publishing LLC, Treasure Island (FL)

[CR96] Yoshikawa A (2015). Deletion of Atf6α impairs astroglial activation and enhances neuronal death following brain ischemia in mice. J Neurochem.

[CR97] Zamanian JL, Xu L, Foo LC, Nouri N, Zhou L, Giffard RG, Barres BA (2012). Genomic analysis of reactive astrogliosis. J Neurosci.

[CR98] Zhang Y, Barres BA (2010). Astrocyte heterogeneity: an underappreciated topic in neurobiology. Curr Opin Neurobiol.

